# Comprehensive Evaluation of Factors Affecting Tremor Relapse after MRgFUS Thalamotomy: A Case-Control Study

**DOI:** 10.3390/brainsci11091183

**Published:** 2021-09-09

**Authors:** Federico Bruno, Alessia Catalucci, Francesco Arrigoni, Alessio Gagliardi, Elena Campanozzi, Antonella Corridore, Emanuele Tommasino, Valeria Pagliei, Leonardo Pertici, Pierpaolo Palumbo, Patrizia Sucapane, Davide Cerone, Francesca Pistoia, Ernesto Di Cesare, Antonio Barile, Alessandro Ricci, Carmine Marini, Alessandra Splendiani, Carlo Masciocchi

**Affiliations:** 1 Department of Biotechnological and Applied Clinical Sciences, University of L’Aquila, 67100 L’Aquila, Italy; arrigoni.francesco@gmail.com (F.A.); gagliardialessio87@gmail.com (A.G.); elenacampanozzi@libero.it (E.C.); antonella.corridore@gmail.com (A.C.); emanuele.tommasino@gmail.com (E.T.); valeria.pagliei@gmail.com (V.P.); leonardo.pertici@gmail.com (L.P.); francesca.pistoia@univaq.it (F.P.); antonio.barile@univaq.it (A.B.); carmine.marini@univaq.it (C.M.); alessandra.splendiani@univaq.it (A.S.); carlo.masciocchi@univaq.it (C.M.); 2 Italian Society of Medical and Interventional Radiology (SIRM), SIRM Foundation, 20122 Milan, Italy; palumbopierpaolo89@gmail.com; 3 Neuroradiology and Interventional Radiology, San Salvatore Hospital, 67100 L’Aquila, Italy; alessiacat@tiscali.it (A.C.); ernesto.dicesare@univaq.it (E.D.C.); 4 Neurology, San Salvatore Hospital, 67100 L’Aquila, Italy; p_sucapane@yahoo.com (P.S.); davide.cerone@yahoo.it (D.C.); 5 Neurosurgery, San Salvatore Hospital, 67100 L’Aquila, Italy; alex.ricci@email.it

**Keywords:** tremor, Parkinson’s disease, MRgFUS thalamotomy, DTI

## Abstract

Objective: To identify possible relevant factors contributing to tremor relapse after MRgFUS thalamotomy in patients with essential tremor (ET) and Parkinson’s disease (PD). Methods: We identified patients with tremor relapse from a series of 79 treatments in a single institution. The demographic and clinical characteristics of the study group patients were compared to those of patients who did not relapse in the same follow-up period. Imaging and procedural factors were compared using a control group matched for clinical and demographic characteristics. Results: Concerning clinical and demographic characteristics, we did not find statistically significant differences in gender and age. Seventy-three percent of patients with tremor relapse were Parkinson’s disease patients. Using MRI, we found larger thalamotomy lesions at the 1-year follow-up in the control group with stable outcomes, compared to patients with tremor relapse. In the tractography evaluation, we found a more frequent eccentric position of the DRTt in patients with tremor relapse. Conclusions: The most relevant determining factors for tremor relapse after MRgFUS thalamotomy appear to be tremor from Parkinson’s disease and inaccurate thalamic targeting. Size of the thalamotomy lesion can also influence the outcome of treatment.

## 1. Introduction

Over recent years, Magnetic Resonance-guided Focused Ultrasound (MRgFUS) thalamotomy has become an attractive treatment for medically intractable tremor due to Essential Tremor (ET) or Parkinson’s Disease (PD); the technique shows clinical effectiveness with minimal invasiveness compared to other treatments [1,2,3,4,5,6]. With accumulating experience and research, many critical aspects of MRgFUS have significantly evolved, namely patient selection criteria, surgical targeting, and technique optimization [7,8,9,10,11,12]. In addition, a growing number of studies using long-term follow-up have focused on the possibility of relapse after treatment. The average tremor recurrence rate after thalamotomy, reported in the literature, is expected to be around 11% within six months post-surgery, although tremor severity is generally less disabling than baseline [13,14,15,16]. A study by Kim et al. reported tremor recurrence returning to baseline tremor severity in 4.3% of cases [17]. Some of the experiences published so far have explored the correlation between tremor improvement and certain technical and patient-related factors, trying to weight the impact of these factors on tremor improvement [13,17,18,19]. An important role is also recognized in MRI imaging, both in terms of treatment planning and potential prognostic value [12,20].

However, there are no studies that comprehensively analyze the impact of various factors, related to both the patient and treatment, capable of explaining the onset of tremor relapse during follow-up. Therefore, our study aimed to evaluate the influence of demographic, clinical, procedural, and imaging parameters on the clinical outcomes of patients submitted to MRgFUS Vim thalamotomy, compared to those with sustained, optimal outcomes.

## 2. Materials and Methods

We retrospectively evaluated all patients submitted to MRgFUS Vim thalamotomy at our Institution between March 2018 and January 2021. From clinical reports, we retrieved patients with tremor relapse (defined as an increase in the Fahn–Tolosa–Marin FTM score of >5 points in any of the follow-up visits after the post-procedural clinical assessment). In accordance with our protocol, all patients were subjected to clinical and instrumental follow-up at one day, one month, six months, and one year after treatment.

Patients who completed clinical and imaging follow-up after treatment for at least one year were included in the study.

In all patients, we recorded clinical–demographic features, procedural data, and imaging findings. Patients with missing or incomplete clinical data, procedural reports, and MRI follow-up were excluded.

Clinical and demographic characteristics included: underlying pathology, age, gender, disease duration, and SDR (skull density ratio). Clinical and demographic characteristics were compared to a control group of 41 patients, drawn from the total population of treated patients, who did not have tremor relapse at the same follow-up intervals.

### 2.1. Procedural Data Were Retrieved from Procedural Reports and Included

- Skull area (cm^2^), i.e., the cranial surface available to be crossed by active transducers (lower cut-off value 350 cm^2^);
- Accumulated thermal dose (ATD) temperature, i.e., the average temperature accumulated during treatment sonications. This was measured by manually placed ROIs on the last sonication heat map, as described in previous studies [10,13];
- Accumulated thermal dose (ATD) area, i.e., the size (mm^2^) of the average temperature accumulated during treatment sonications. This was measured by manually placed ROIs on the last sonication heat map, as described in previous studies [10,13];
- Active elements, i.e., the number of active transducers (lower cut-off value: 700)
- Sonications, i.e., the total number of sonications performed during the treatment;
- Target movements, i.e., the number of target coordinate shifts performed during the treatment;
- Maximum power (Watt), i.e., the maximum power level set during sonications;
- Maximum energy (Joule), i.e., the maximum energy level set during sonications;
- Mean temperature (°C), i.e., the highest value of mean temperature reached during sonications;
- Maximum sonication duration, i.e., duration of each sonication expressed in seconds.

For the evaluation of procedural parameters we selected a control group of patients, who did not experience tremor relapse, from the cohort of all patients treated. Control patients were matched pairwise for age, sex, pathology, years of disease, pre-treatment FTM score, and SDR values (Table 1).

### 2.2. Imaging Evaluation Included

**Figure 1:**
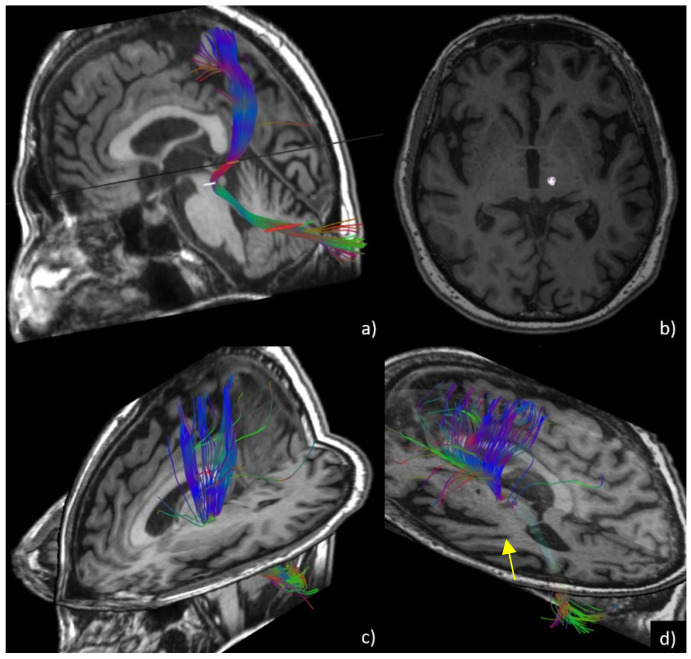
DTI tractography of the DRTt (dentato-rubro-thalamic tract). In (**a**), 3D reconstruction of the fiber bundle is shown through ROI seeding, superimposed on the volumetric T1 sequence at the levels of the dentate nucleus, red nucleus, and thalamus. In (**b**), ROI position of the DRTt at the level of the thalamus is shown, for quantitative analysis of ADC and FA values. In (**c**) and (**d**), central/eccentric location of the DRTt is shown. The bundle is centered within the lesion in c while appearing eccentric with respect to the hypointense thalamotomy lesion (arrow) in (**d**).

- Measurement of the lesion size at the thalamus level, expressed in millimeters, measured as the maximum diameter of T2-weighted sequences in the axial plane. All examinations were performed using a 3-Tesla MR scanner (MR750w, GE Healthcare) with a 32-channel head coil. Acquisition parameters were: slice 3.0–0.3, TR 7854, freq. FOV 26, phase FOV 0.8. The same MRI protocol was applied for the follow-up examinations at 24 h, one month, six months, and 12 months after treatment. Thalamotomy lesions were manually measured on a PACS workstation (Vuemotion, Carestream Health) by two neuroradiologists (A.C., F.B., with 16 and 4 years of experience in neuroimaging, respectively) using a digital ruler tool. The slice at the thalamus level that showed the greatest extent of the lesion and edema was chosen.
- Tractography evaluation of the dentato-rubro-thalamic tract (DRTt) before and six months after treatment. DTI sequences were acquired using the following parameters: 33 diffusion directions, TR 5700 ms, TE 98 ms, parallel imaging (acceleration factor two), 3 mm slice thickness, 39 slices, matrix 128 × 128, 230 mm FOV, b value 1000 s/mm^2^, acquisition time 4:01 min. A T1-weighted 3D IR FSPGR BRAVO sequence with multiplanar reconstructions was also acquired (parameters: FOV 24, slice thickness 1.6 mm, flip angle 20°, prep time 450, TE 3.2, matrix 256 × 192, NEX 3, duration 13 min). Probabilistic fiber tracking was performed using a dedicated software (Brainance MD, Advantis Medical Imaging, Eindhoven, NL). An EPI correction tool for distortion correction was applied before image analysis. The fractional anisotropy threshold was set at 0.15, minimum fiber length 0 mm, maximum fiber length 200 mm, angular threshold 27°, and step size 1 mm. The dentato-rubro-thalamic tract (DRTt) was obtained by manual definition of the following three regions of interest (ROIs) on axial images, as described in previous experiments [21]: the cerebellar dentate nucleus ipsilateral to the target, the ipsilateral red nucleus, and the supposed location of the ipsilateral Vim at the level of the thalamus on the AC-PC plane. We evaluated whether the bundle was eccentric or central with respect to the thalamotomy lesion using post-procedural images six months after treatment. Similar to the methods described by Miller et al. [9], the two neuroradiologists measured the amount of overlap between the thalamotomy lesions and the DRTts and classified the bundle position as central (overlap > 50%) or eccentric (overlap < 50%). Moreover, ADC and FA values at the thalamotomy level were measured using the ROI previously set for DRTt tractography (Figure 1).

Imaging analysis comparisons were carried out in the same matched control group selected for procedure-related parameters.

### 2.3. Statistical Analysis

Data analyses were performed using XLSTAT 2017: Data Analysis and Statistical Solution for Microsoft Excel (Addinsoft, Paris, France 2017). Qualitative variables were summarized as frequency and proportions. Values of continuous variables were tested for normal distribution using Shapiro–Wilk’s test and reported as means and standard deviations (SD) or medians and interquartile ranges (IQR) according to their distribution. Differences of quantitative values (age, disease duration, SDR, skull area, ATD area, ATD temperature, elements, sonications, power, target movements, energy, temperature, sonication duration, and lesion size) between groups were compared using the Wilcoxon test. The Kruskal–Wallis test was applied to evaluate variance differences between groups (ADC, FA). Fisher’s test was applied to compare differences between groups of binomial data (underlying pathology, sex, DRTt location).

## 3. Results

### 3.1. Clinical and Demographic Parameters

Out of a total of 79 patients treated during the study period, 11 patients (eight males, three females; mean age 61.8 ± 9.26, range 47–74; mean disease duration of 9.9 ± 5.57 years) showed evidence of tremor relapse during the follow-up. Of these, 72% were PD patients, and 27% were ET patients.

**Figure 2:**
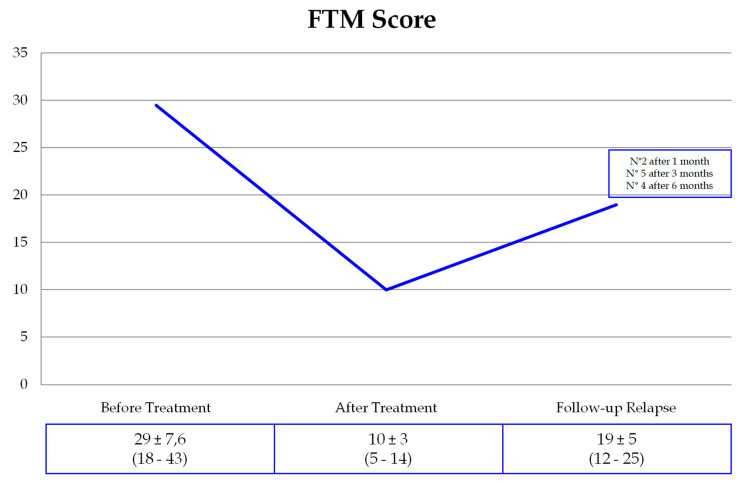
FTM scores in the study group.

Two patients experienced tremor recurrence one month after treatment, five patients after three months, and four patients after six months. Mean FTM tremor relapse scores were lower than baseline in all patients (Figure 2).

The control group was composed of 41 patients (18 PD, 23 ET, 10 females, 31 males) with a mean age of 67 ± 10.4 years and a mean disease duration of 16.9 ± 8.5 years.

Patients in the study group showed significantly higher SDR values compared with the control group. The statistical analysis did not show any significant differences between the study group and control group in gender (chi-square 0.035, *p*-value 0.84) and age (Table 2).

#### 3.1.1. Procedural Parameters

Concerning the analysis of procedural data, we found statistically significant lower values for power, energy, and sonication duration in the study group, and higher values for ATD area and maximum temperature, compared to the control group. In patients with relapse, a significantly higher number of target movements were also performed during treatment. No significant differences were found between the two groups in terms of skull area, ATD temperature, number of active elements, and number of sonications.

Detailed comparisons between the procedural parameter values of patients in the relapse and control groups are summarized in Table 3.

#### 3.1.2. Imaging Parameters

In both groups, we found a progressive decrease in the thalamotomy lesion size (Table 4). According to MRI at one year follow-up, the study (relapse) group patients had lesions that were significantly smaller when compared to those of patients in the control group (*p* = 0.003).

In patients with relapse, the thalamotomy lesion/DRTt overlap after treatment was central in 55% of cases and eccentric in 45% of cases (chi-square 1.69, *p*-value 0.19) (Table 5).

ADC and FA values before and after treatment are summarized in Table 6.

## 4. Discussion

MRgFUS thalamotomy represents an innovative and minimally invasive technique for the treatment of tremors. Compared to other functional neurosurgery procedures (namely, DBS) it is a lesional technique involving the thermal ablation of the ventral intermediate (Vim) nucleus. Despite this, and though there are still few long-term studies, tremor relapse is reported in the literature at a rate of approximately 10–11% [1,7].

The mechanisms underlying the reorganization of motor circuits after thalamotomy are very complex, as are the numerous possible clinical–demographic and technical factors that can influence the procedure’s outcome [17,22,23].

Our study aims to analyze these factors comprehensively and, to the best of our knowledge, is the first study conducted using a case–control design.

### 4.1. Clinical and Demographic Data

In our sample, 11 patients with tremor relapse were primarily composed of patients with PD; a minority of patients had ET. These results reflect those of Schlesinger et al. who suggested that tremor recurrence is more frequent in patients affected by PD or those with long-standing ET prior to developing PD symptoms [16,24]. While this is particularly true for Vim ablation, it appears that targeting the subthalamus or globus pallidus may have a better outcome in Parkinson’s patients [14,15].

In our study, no significant differences regarding patients’ age on the clinical outcome emerged, whereas patients with relapse had a significantly shorter disease duration. In a previous study of 179 ET patients conducted by Krishna et al., younger age and shorter disease duration were statistically significant predictors of hand tremor improvement [17]. These results emerged from a multivariate analysis of all treated patients. Our methods evaluated the outcomes of patients with tremor relapse by comparing them with patients with stable tremor improvement. In contrast to their findings, our results revealed that subjects with a longer history of disease showed fewer relapses. This could be linked to most of these patients having ET and the fact that ET patients usually have a longer disease history at the time of presenting for treatment compared to PD patients. Notably, in a previous metanalysis of outcomes following subthalamic nucleus (STN) deep brain stimulation (DBS), the authors documented more remarkable changes in UPDRS scores in cases of increased disease duration prior to surgery.

Furthermore, consistent with previous literature results, no statistically significant differences in patients’ outcomes emerged as a result of patients’ genders [25].

### 4.2. Technical and Procedural Parameters

The procedural aspects of FUS (focused ultrasound surgery) are based on the setting and management of ultrasound waves’ physical parameters, namely energy, power, and duration of sonication [26]. For transcranial FUS procedures, the skull density ratio (SDR) is an essential index for evaluating to what extent the skull can be a barrier to focused ultrasound traversing through the brain. In fact, the skull distorts the ultrasound waves, absorbs energy—leading to skull heating—and attenuates the ultrasound beam [18,19,23,27]. In our cohort, patients with relapse showed a significantly higher SDR than controls. Nevertheless, these data do not indicate that higher SDR values are associated with a worse outcome, but rather that, despite low SDR values, it is still possible to obtain an excellent therapeutic result by modulating technical parameters. Kung Won Chang et al., in a study on 318 patients with a mean SDR of 0.45, described a non-statistically significant influence of both SDR values and skull area on treatment outcome, underlining a relationship of the SDR with head volume and patient sex, but not with age. This is explained by considering the influence of low SDR values on procedural parameters and the technical complexity of the treatment but not its effectiveness, evidence that has already emerged in previous literature studies [18,19].

The ATD area also seems to be linked to patient outcome. According to our results, higher ATD area values were found in patients with relapse; this finding contrasts with some literature data. Using a sample of eight patients followed up over one year, Federau et al. [23] highlighted a correlation between the volume of accumulated thermal dose, the volume of the lesion, and the clinical subscore, arguing that the dose can represent a tool for monitoring treatment and its effectiveness. Furthermore, they defined the need to have a minimum ATD area of 76 mm^3^ to obtain a final lesion of at least 36 mm^3^ and guarantee adequate clinical improvement. In our experiment, thalamotomy lesions had an ATD area higher than these cut-offs in all patients.

The number of sonications was not significantly different between patients with relapse and the control group. This result contrasts with that of Krishna et al. [17] who found an association between negative outcomes and the use of a greater number of sonications, probably linked to a possible increase in power requirements with multiple sonications.

Power, energy, and sonication duration were significantly different in patients with and without tremor relapse. However, as previously discussed, these data are likely related to the lower SDR scores of patients included in the control group and the consequent adjustment to technical parameters required during the treatment to reach ablative temperatures.

In patients with relapse, we found that higher mean temperature peaks were reached during treatment than those observed in the control group. This finding contradicts previous studies that have suggested that better results are associated with the achievement of higher temperatures [17]. However, this may indeed be likely since ablative temperatures have always been reached, while, on the contrary, recurrence is predictable in cases where temperatures are not ablative (i.e., <56 °C).

While no differences in the lesion sizes between the two groups were demonstrated at the one-day, one-month, and six-month MRI follow-ups, we found significantly larger lesions at the 1-year control in patients without tremor relapse. This agrees with the study by Jeffrey D. Atkinson et al. [28] in which patients with excellent post-treatment results presented larger lesions. In any case, according again to this study, the best predictor would be represented by an adequate targeting of the area subjected to ablation.

Finally, the number of target shifts during treatment was significatively higher in patients with tremor recurrence, which could also imply an influence on the outcome of these patients. Indeed, the main reason for target shift during treatment is a non-optimal clinical response with subablative sonication at the verification stage (i.e., reaching temperatures up to 50–55 °C), likely reflecting an incorrect targeting [21].

### 4.3. Imaging Findings

Although the Vim is a small thalamic nucleus, substantially invisible to imaging even using advanced MR sequences, and indirect targeting using stereotactic coordinates still represents the gold-standard approach, MR imaging has a non-marginal role in the planning and follow-up of patients undergoing MRgFUS thalamotomy [20,28,29]. Tractography of the dentato-rubro-thalamic fibers using DTI sequencing is recognized as a valid method for directly identifying the treatment target at the level of the thalamus, and its interruption was demonstrated to be associated with clinical improvement [21,22]. Chazen et al. [22] previously demonstrated the reliability of DTI in identifying the optimal ablation target with the dentato-rubro-thalamic tract. Miller et al. also showed the overlap of the thalamotomy lesion with the DRTt bundles in patients with tremor relief after MRgFUS thalamotomy [9]. In our experience [21], we also compared direct and indirect targeting of the Vim, finding inferior error values on the RL and AP coordinates using DRTt tractography. In our study, we found a significantly lower rate of overlap between the thalamotomy lesion and the tractography-reconstructed dentato-rubro-thalamic tract in patients with tremor relapse, confirming the non-optimal target centering (Figure 1).

There were no significant differences in ADC values before and after treatment between the two groups. In all cases, the average FA values were reduced after treatment, in line with reports from the previous scientific literature [30]. These findings reflect the loss of axonal integrity determined in the lesion region. Notably, in the control group, FA values tended towards a greater reduction (though without reaching statistical significance), maybe reflecting a more complete disruption of the fiber tracts.

The present study possessed some limitations that deserve mention. First of all, the study group was composed of a limited number of participants given that the relapse rate after MRgFUS thalamotomy was relatively low. We also only had follow-ups available at one year. However, this latter aspect seemed to have minimal impact, given that all relapses appeared within six months of treatment. Further studies with a larger population and a longer follow-up period may be needed to corroborate our observations.

## 5. Conclusions

Tremor relapse after MRgFUS thalamotomy can occur in a limited percentage of patients. In our study, the most relevant determining factors for tremor relapse after MRgFUS thalamotomy appeared to be the presence of Parkinsonian tremor and inaccurate thalamic targeting. The size of the thalamotomy lesion can influence the outcome of the treatment.

## Figures and Tables

**Table 1:** Summary characteristics of study and control group for the comparison of procedural parameters.

	Study Group	Control Group
**sex (m/f)**	8/3	8/3
**pathology (et/pd)**	3/8	3/8
**disease duration**	11.72 ± 8.03 (3–30)	18.81 ± 7.44 (2–39)
**age**	61.81 ± 9.26 (47–74)	67.36 ± 8.26 (57–78)
**ftm**	29.54 ± 7.96 (18–43)	29.63 ± 11.53 (6–48)
**sdr**	0.47 ± 0.06 (0.35–0.57)	0.43 ± 0.08 (0.27–0.57)

**Table 2:** Summary of clinical and demographic parameters in the relapse study group and control group. Statistically significant values (*p* < 0.05) are in bold.

Clinical Features	Study Group	Control Group	*p*-Value
pd	8 (72.7%)	18 (43.7%)	**<0.05**
et	3 (27.3%)	23 (56.3%)	
Age (Years)	61.8 ± 9.26 (47–74)	67.1 ± 10.49 (48–85)	0.144
Gender	3 F; 8 M	10 F; 31 M	0.84
Disease Duration (Years)	9.9 ± 5.57 (3–19)	16.94 ± 8.53 (2–31)	**0.022**
sdr	0.48 ± 0.06 (0.35–0.57)	0.43 ± 0.09 (0.27–0.57)	**0.001**

**Table 3:** Procedural parameters in the study group and control group.

	Study Group	Control Group	*p*-Value
**Skull Area** **(mm^2^)**	330 ± 22.73 (302–384)	327.64 ± 32.29 (289–378)	0.979
**Accumulated Thermal Dose (ATD) Area** **(mm^2^)**	26.54 ± 15.35 (6–50)	16.18 ± 12.58 (1–41)	0.001
**Accumulated Thermal Dose (ATD) Temperature** **(°C)**	54.18 ± 2.2 (51–58)	54.09 ± 2.35 (49–57)	0.689
**Elements** **(*n*)**	903 ± 53.07 (809–984)	893 ± 29.09 (835–939)	0.171
**Sonications** **(*n*)**	12.82 ± 3.35 (7–19)	12.27 ± 3.49 (7–18)	0.715
**Power** **(W)**	753.73 ± 128.76 (453–990)	848 ± 132.51 (651–1100)	0.002
**Target Movements**	2.09 ± 2.47 (0–7)	1.18 ± 1.61 (0–5)	0.035
**Energy** **(J)**	12,492.46 ± 5791.50 (5200–26,000)	17,821.46 ± 7935.42 (8337–31,700)	0.009
**Temperature** **(°C)**	62.82 ± 2.93 (58–68)	59.91 ± 3.14 (56–64)	0.004
**Sonication Duration** **(s)**	20.62 ± 7.78 (13–35)	28 ± 12.88 (13–50)	0.042

**Table 4:** Thalamotomy lesion size (mm).

	1 Day	1 Month	6 Months	1 Year
Study Group	7.68 ± 1.02	6.85 ± 1.60	4.12 ± 0.72	4.33 ± 0.51
Control Group	7.81 ± 1.61	6.79 ± 2.59	6.21 ± 1.01	5.99 ± 1.48
*p*-value	0.478	0.773	0.083	0.003

**Table 5:** Comparison of DRTt location distribution between the study and control group.

	Study Group	Control Group	*p* Value
Central	55.56%	72.73%	0.017
Eccentric	44.44%	27.27%	

**Table 6:** ADC and FA values before and after treatment in the study and control group.

	ADC Before Treatment	ADC After Treatment	FA Before Treatment	FA After Treatment
Study Group	8.11 × 10^−4^ ± 2.8 × 10^−4^ (4.77 × 10^−4^–1.38 × 10^−3^)	6.9 × 10^−4^ ± 1.04 × 10^−4^ (5.74 × 10^−4^–8.59 × 10^−4^)	0.41 ± 0.11 (0.28–0.65)	0.20 ± 0.08 (0.08–0.34)
Control Group	7.27 × 10^−4^ ± 6.56 × 10^−5^ (5.99–8.02 × 10^−4^)	7.05 × 10^−4^ ± 8.59 × 10^−5^ (6.09–8.74 × 10^−4^)	0.38 ± 0.09 (0.24–0.51)	0.21 ± 0.09 (0.10–0.39)
*p*-value	0.667	0.811	0.861	0.826

## Data Availability

The data supporting the findings of this study are available from the corresponding author upon reasonable request.
